# Supplementary material for: Gut Microbiota Mediates the Protective Effects of Traditional Chinese Medicine Formula Qiong-Yu-Gao against Cisplatin-Induced Acute Kidney Injury
Source: Microbiol Spectr. 2022 Apr 28;10(3):e00759-22. doi: 10.1128/spectrum.00759-22 (PMC9241845; doi:10.1128/spectrum.00759-22)
Supplement: SUPPLEMENTAL FILE 1 — Supplemental material. Download spectrum.00759-22-s001.pdf, PDF file, 1.8 MB [file spectrum.00759-22-s001.pdf]

Supporting Information for

Gut microbiota mediates the protective effects of traditional Chinese medicine formula Qiong-Yu-Gao against cisplatin-induced acute kidney injury

Ye-Ting Zou[a,b], Jing Zhou[a], Jin-Hao Zhu[a,b], Cheng-Ying Wu[a], Hong Shen[b], Wei Zhang[a], Shan-Shan Zhou[b], Jin-Di Xu[a], Qian Mao[b], Ye-Qing Zhang[c], Fang Long[a,c,#], Song-Lin Li[a,b,#]

[a]*Department of Pharmaceutical Analysis, Affiliated Hospital of Integrated Traditional Chinese and Western Medicine, Nanjing University of Chinese Medicine, Nanjing, China.*

[b]*Department of Metabolomics, Jiangsu Province Academy of Traditional Chinese Medicine, Nanjing, China.*

[c]*Department of Respiratory Medicine, Affiliated Hospital of Integrated Traditional Chinese and Western Medicine, Nanjing University of Chinese Medicine, Nanjing, China.*

Running Head: Gut microbiota mediates nephroprotective effects of Qiong-Yu-Gao

#Address correspondence to Song-Lin Li, songlinli64@126.com, or Fang Long, longfangcpu@163.com.

Supplementary materials and methods

1. Characterization of prepared QYG samples

1.1. molecular weight distribution of polysaccharide

For chemical profiling of QYG, the prepared samples were diluted with 5-fold of water, and subsequently precipitated by adding ethanol to a final concentration of 75% (v/v), left overnight at 4 °C. Lyophilized residue polysaccharide (40 mg) was dissolved in 5 mL of water, and submitted to HPGPC equipped with a TSK-gel G4000PWXL column (300 mm×7.8 mm i.d., 10 μm) under a constant flow (0.5 mL/min) at 35 °C. The column temperature was 35 °C. The injection volume of sample was 20 μL. The elute was monitored by an evaporative light-scattering detector with nitrogen as nebulizer gas (pressure: 40 psi). the drift tube temperature was 90 °C. The molecular weight distribution was estimated by the calibration curve made from a series of Dextran reference (1-2000 kDa, Fig. S4).

1.2. Monosaccharide composition of polysaccharide

Accurately weighed 40 mg of lyophilized residue polysaccharide in 3 mL of trifluoroacetic acid (2 mol/L) was heated at 120 °C for 2 h to hydrolysis. The resulting solution was then evaporated and subjected to derivatization by 1-phenyl-3-methyl-5-pyrazolne (PMP). After derivatization, PMP derivatives of monosaccharides were analysed by HPLC using Grace AlltimaTM C18 column (250 mm×7.8 mm i.d., 5 μm) with a flow rate of 1 mL/min. The column temperature was maintained at 35 °C and the

wavelength of the UV detector was set at 245 nm. The mobile phase consisted of (A) acetonitrile and (B) 0.1 mol/L ammonium acetate solution. A linear gradient elution program was set as follows: 0–8 min, 16–20% A; 8–25 min, 20–25% A; 25–27 min, 25–90% A. The injection volume of sample was 10 μL. The monosaccharide composition was determined by comparing with the standard monosaccharides derivatized with PMP (Fig. S5).

1.3. Characterization of the supernatant

Accurately weighed 65 mg of lyophilized supernatant was ultrasonic-extracted with 10 mL of methanol for 30 min. Then, the extracts were filtered through 0.2 μm PTFE syringe and 2.0 μL of filtrates were subjected to UPLC-QTOF-MS/MS analysis. Liquid chromatographic separation was performed on a Waters ACQUITY UPLC™ system (Waters, MA, USA) equipped with an ACQUITY UPLC HSS T3 column (2.1 × 100 mm, 1.8 μm). The column temperature was held at 40 °C during the analysis. Acetonitrile (A) and ultrapure water (B), both containing 0.1% formic acid (v/v) were used as the mobile phase. The metabolites were eluted at flow rate of 0.4 mL·min-1 with the following optimal gradient program: 0–0.5 min, 1% A; 0.5–3.5 min, 1–15% A; 3.5–15 min, 15–35% A; 15–20 min, 35–40% A; 20–22 min, 40–60% A; 22–27 min, 60–80% A. A Waters Q-TOF Synapt G2 mass spectrometer (Waters MS Technologies, Manchester, UK) was used for mass detection, and the operation parameters were set as follows: capillary voltage 2.5 kV; cone voltage 40 V; cone gas flow rate 50 L/h; source temperature 100 °C; desolvation temperature 450 °C; desolvation gas flow rate

800 L/h. The scan mass range was 100–1200 Da. In MS^E mode, the trap collision energy of the low energy function was set at 6 eV, and the high-energy function was ramped from 30 to 60 eV. Components in the supernatant were identified based on our previously established methods (Fig. S6 and Table S1).

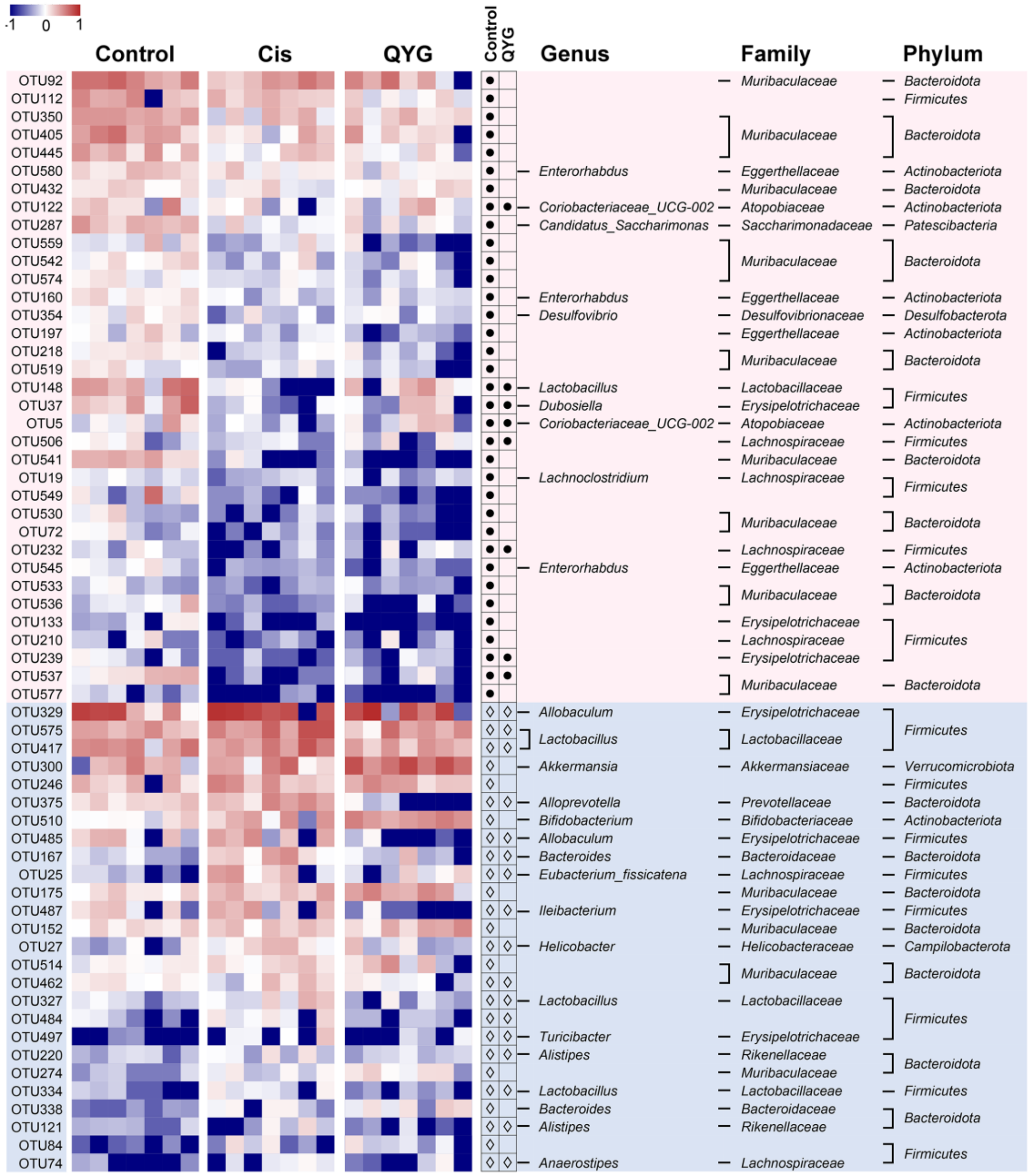

FIG S1 Heatmap of key OTUs responsible for cisplatin-induced bacterial composition change. In the middle panel, black dots represent more abundant OTUs in control and QYG groups compared with Cis group; white diamonds represent less abundant OTUs in control and QYG groups compared with Cis group. Bacterial taxa information (genus, family and phylum) of the OTUs is shown on the right panel.

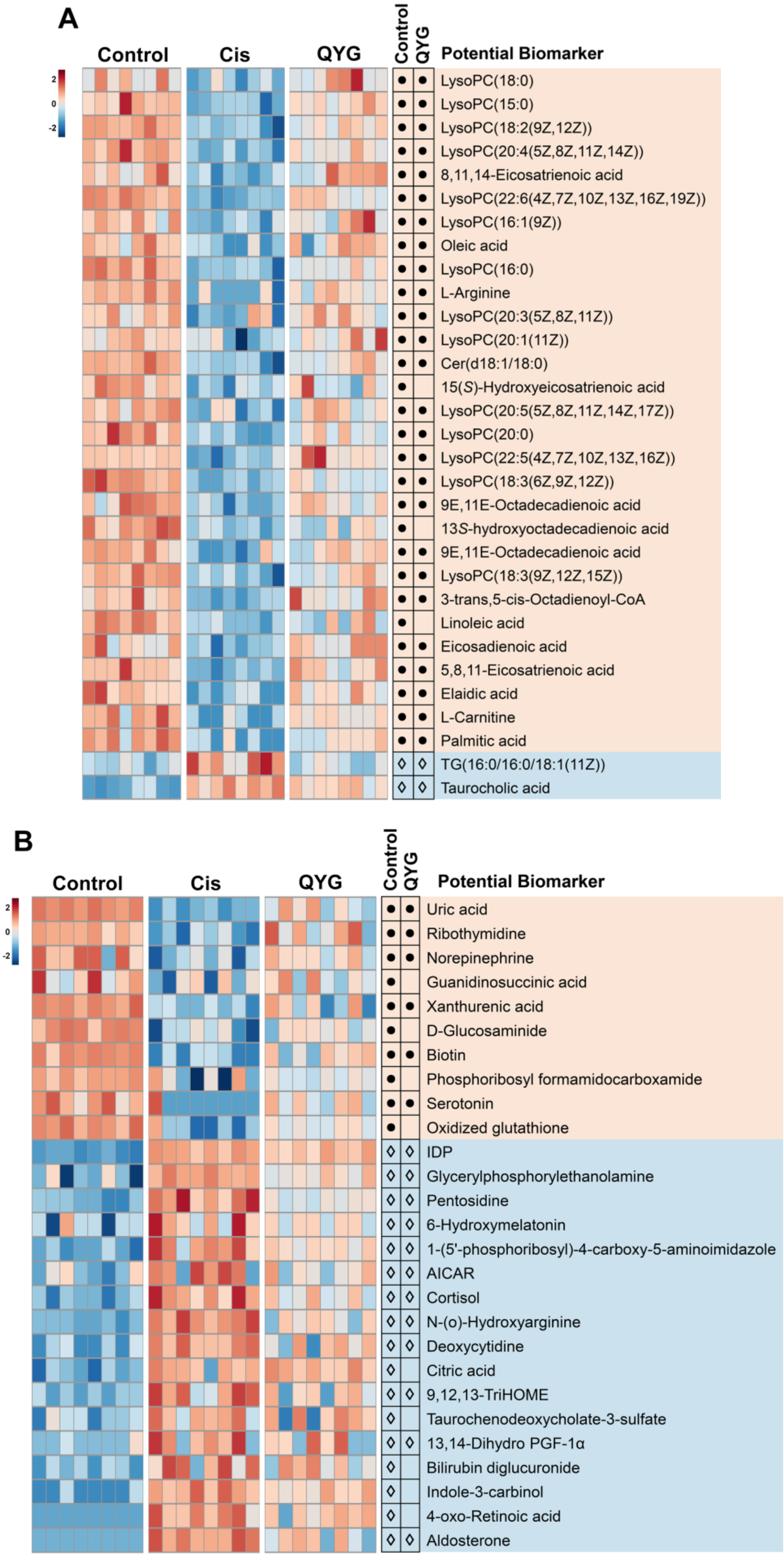
A
Control
Cis
QYG
Control
QYG
Potential Biomarker
2
0
-2
LysoPC(18:0)
LysoPC(15:0)
LysoPC(18:2(9Z,12Z))
LysoPC(20:4(5Z,8Z,11Z,14Z))
8,11,14-Eicosatrienoic acid
LysoPC(22:6(4Z,7Z,10Z,13Z,16Z,19Z))
LysoPC(16:1(9Z))
Oleic acid
LysoPC(16:0)
L-Arginine
LysoPC(20:3(5Z,8Z,11Z))
LysoPC(20:1(11Z))
Cer(d18:1/18:0)
15(S)-Hydroxyeicosatrienoic acid
LysoPC(20:5(5Z,8Z,11Z,14Z,17Z))
LysoPC(20:0)
LysoPC(22:5(4Z,7Z,10Z,13Z,16Z))
LysoPC(18:3(6Z,9Z,12Z))
9E,11E-Octadecadienoic acid
13S-hydroxyoctadecadienoic acid
9E,11E-Octadecadienoic acid
LysoPC(18:3(9Z,12Z,15Z))
3-trans,5-cis-Octadienoyl-CoA
Linoleic acid
Eicosadienoic acid
5,8,11-Eicosatrienoic acid
Elaidic acid
L-Carnitine
Palmitic acid
TG(16:0/16:0/18:1(11Z))
Taurocholic acid
B
Control
Cis
QYG
Control
QYG
Potential Biomarker
2
0
-2
Uric acid
Ribothymidine
Norepinephrine
Guanidinosuccinic acid
Xanthurenic acid
D-Glucosaminide
Biotin
Phosphoribosyl formamidocarboxamide
Serotonin
Oxidized glutathione
IDP
Glycerylphosphorylethanolamine
Pentosidine
6-Hydroxymelatonin
1-(5'-phosphoribosyl)-4-carboxy-5-aminoimidazole
AICAR
Cortisol
N-(o)-Hydroxyarginine
Deoxycytidine
Citric acid
9,12,13-TriHOME
Taurochenodeoxycholate-3-sulfate
13,14-Dihydro PGF-1α
Bilirubin diglucuronide
Indole-3-carbinol
4-oxo-Retinoic acid
Aldosterone

51

Continued

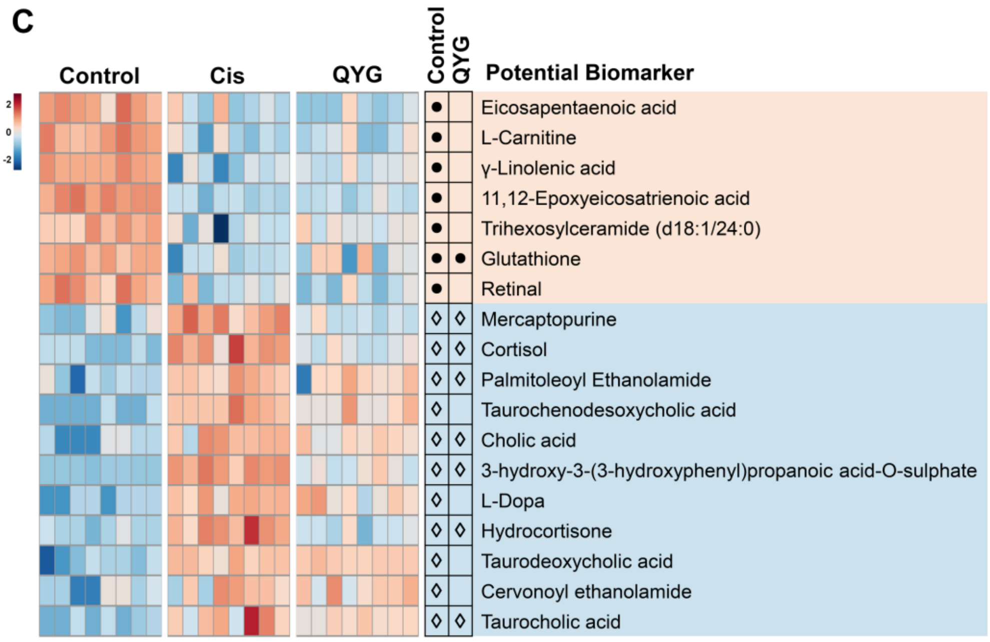

FIG S2 Heatmaps summarizing potential biomarker of cisplatin-induced injury in serum (A), urine (B) and renal tissue (C), and their response to QYG treatment. The color of the spots in the left presents the relative abundance of metabolites in each group. In the middle, the black dot represents more abundant in control and QYG group compared with Cis group, while the the white diamond represents less abundant in control and QYG group compared with Cis group.

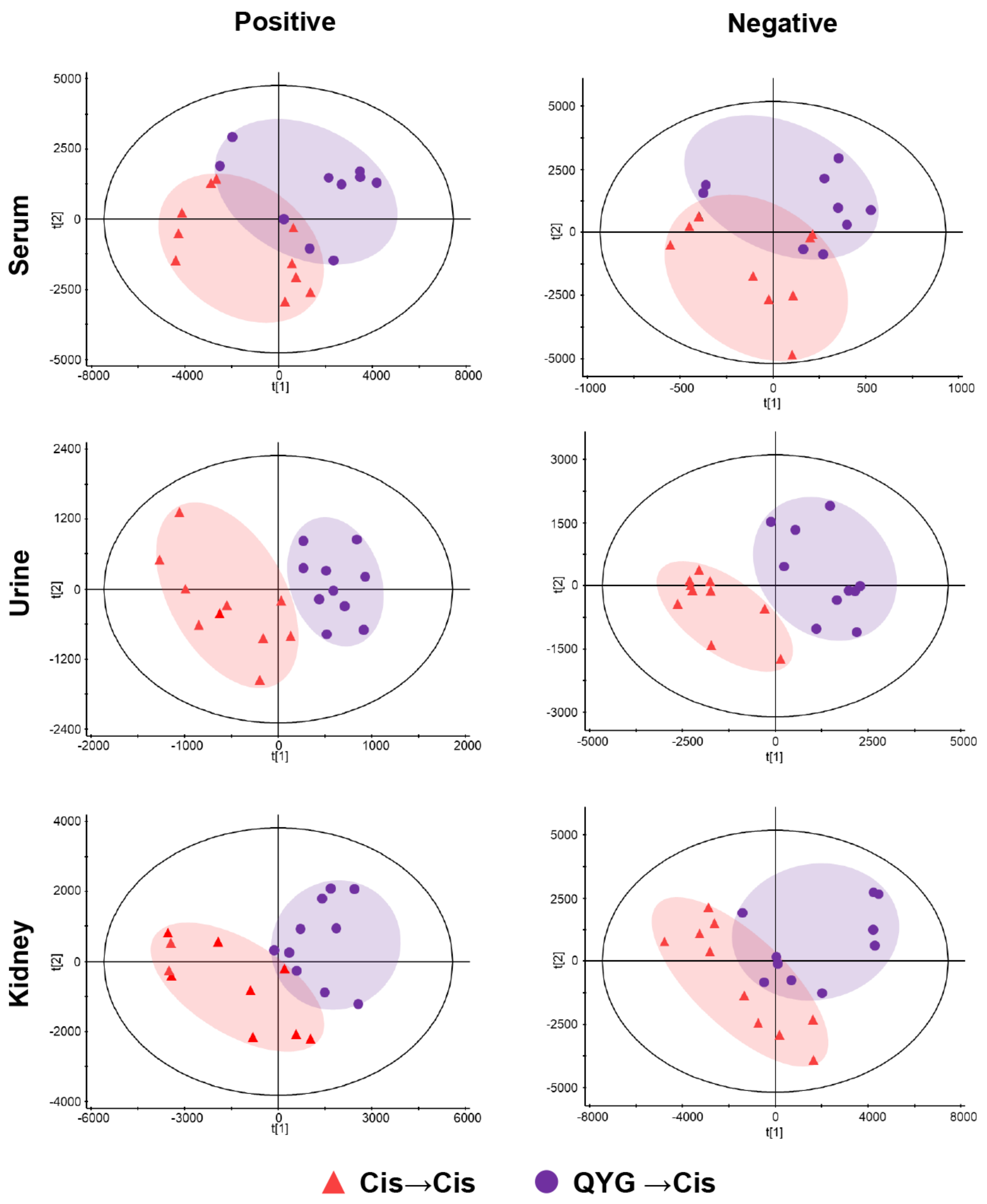

FIG S3 PLS-DA score plots of metabolic profiling of serum, urine and renal tissue in positive and negative ion mode.

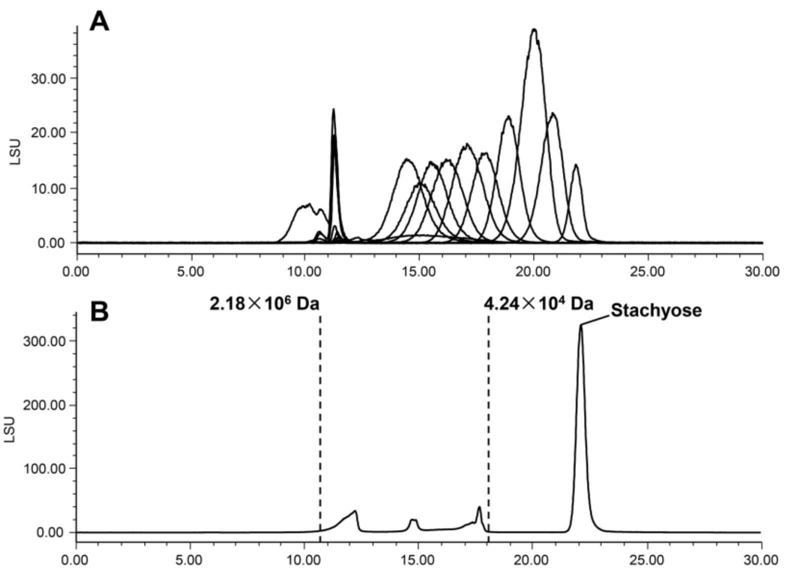

FIG S4 The HPGPC chromatograms of Dextran references (A) and polysaccharides in QYG (B). The HPGPC chromatograms showed that the residue polysaccharides have a wide molecular weight distribution ranged from 4.24×10^4 Da to 2.18×10^6 Da.

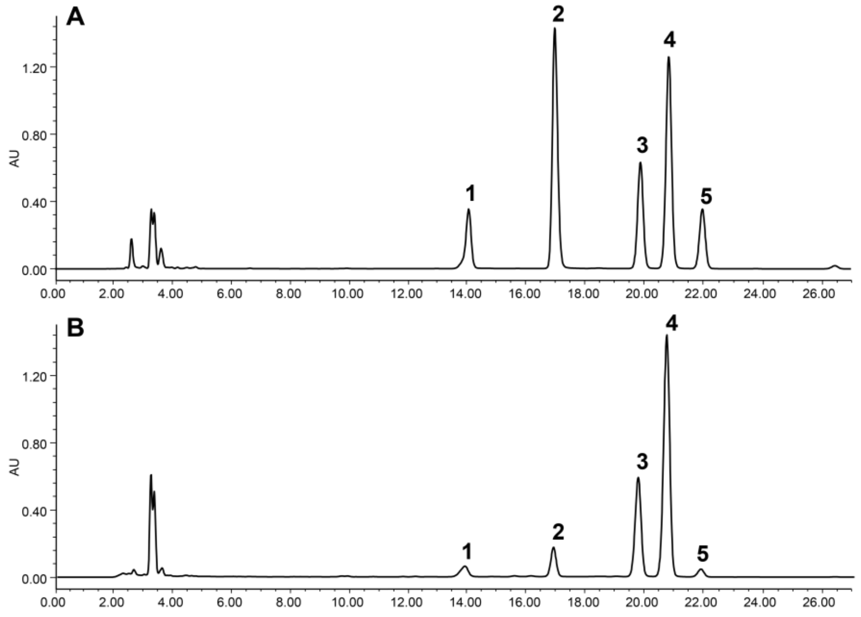

FIG S5 HPLC analysis of standard monosaccharides (A) and hydrolysed polysaccharides in QYG (B) derivatized with PMP. The monosaccharide composition of residue polysaccharides was determined as mannose, galacturonic acid, glucose, galactose and arabinose at a molar ratio of 1.00:4.97:50.19:60.40:3.23. Peaks: 1, mannose; 2, galacturonic acid; 3, glucose; 4, galactose; 5, arabinose.

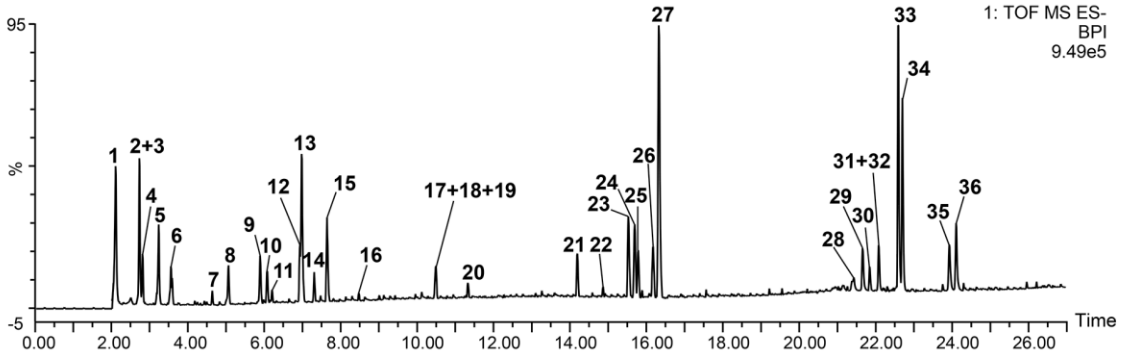

FIG S6 Base peak intensity chromatogram of supernatant analysed by UPLC-QTOF-MS/MS. A total of 36 components were identified, and glycosides including catapol, acteoside, Rg2, Ro, and 20(S/R)-Rg3 were determined to be the main components. Information of 36 identified components is presented in Table S1.

TABLE S1 Components identified from supernatant by UPLC-QTOF-MS/MS.

**No.**	**Identification**	**Formula**	t_R	***m/z***
1	Catapol	$C_{15}H_{22}O_{10}$	2.11	$[M-H+HCOOH]^-$ 407.1185
2	Glutinoside	$C_{15}H_{23}ClO_{10}$	2.73	$[M-H+HCOOH]^-$ 443.0955
3	Rehmannioside D	$C_{27}H_{42}O_{20}$	2.73	$[M-H]^-$ 685.2187
4	Melittoside	$C_{21}H_{32}O_{15}$	2.81	$[M-H+HCOOH]^-$ 569.1722
5	Leonuride	$C_{15}H_{24}O_{9}$	3.23	$[M-H+HCOOH]^-$ 393.1391
6	8-Epiloganic acid/isomer	$C_{16}H_{24}O_{10}$	3.55	$[M-H]^-$ 375.1278
7	Darendoside A	$C_{21}H_{32}O_{12}$	4.64	$[M-H]^-$ 475.1810
8	Echinacoside	$C_{35}H_{46}O_{20}$	5.06	$[M-H]^-$ 785.2517
9	Jionoside A1/Jionoside A2	$C_{36}H_{48}O_{20}$	5.89	$[M-H]^-$ 799.2681
10	Rehmapicrogenin	$C_{10}H_{16}O_{3}$	6.07	$[M-H]^-$ 183.1008
11	Rehmaionoside A/Rehmaionoside B	$C_{19}H_{34}O_{8}$	6.20	$[M-H+HCOOH]^-$ 435.2219
12	Rehmaionoside A/Rehmaionoside B	$C_{19}H_{34}O_{8}$	6.92	$[M-H+HCOOH]^-$ 435.2238
13	Acteoside	$C_{29}H_{36}O_{15}$	6.97	$[M-H]^-$ 623.1989
14	Jionoside B1/Jionoside B2	$C_{37}H_{50}O_{20}$	7.30	$[M-H]^-$ 813.2847
15	Isoacteoside/ Forsythiaside	$C_{29}H_{36}O_{15}$	7.63	$[M-H]^-$ 623.1981
16	Cis-Leucosceptoside A/Leucosceptoside A	$C_{30}H_{38}O_{15}$	8.47	$[M-H]^-$ 637.2141
17	Rg_1	$C_{42}H_{72}O_{14}$	10.46	$[M-H+HCOOH]^-$ 845.4922
18	Martynoside	$C_{31}H_{40}O_{15}$	10.48	$[M-H]^-$ 651.2300
19	Re	$C_{48}H_{82}O_{18}$	10.48	$[M-H+HCOOH]^-$ 991.5514
20	Martynoside isomer	$C_{31}H_{40}O_{15}$	11.32	$[M-H]^-$ 651.2297
21	Rf	$C_{42}H_{72}O_{14}$	14.18	$[M-H+HCOOH]^-$ 845.4933
22	Notoginsenoside R_2	$C_{41}H_{70}O_{13}$	14.85	$[M-H+HCOOH]^-$ 815.4819
23	Rg_2/isomer	$C_{42}H_{72}O_{13}$	15.51	$[M-H+HCOOH]^-$ 829.4984
24	*S*-Rh_1	$C_{36}H_{62}O_{9}$	15.68	$[M-H+HCOOH]^-$ 683.4381
25	*S*-Rg_2	$C_{42}H_{72}O_{13}$	15.77	$[M-H+HCOOH]^-$ 829.4965
26	R-Rh_1	$C_{36}H_{62}O_{9}$	16.16	$[M-H+HCOOH]^-$ 683.4392
27	Ro	$C_{48}H_{76}O_{19}$	16.31	$[M-H]^-$ 955.4956
28	Rg_6	$C_{42}H_{70}O_{12}$	21.40	$[M-H+HCOOH]^-$ 811.4864
29	F4	$C_{42}H_{70}O_{12}$	21.63	$[M-H+HCOOH]^-$ 811.4875
30	Rk3	$C_{36}H_{60}O_{8}$	21.79	$[M-H+HCOOH]^-$ 665.4274
31	Rh_4	$C_{36}H_{60}O_{8}$	22.03	$[M-H+HCOOH]^-$ 665.4273
32	Zingibroside R_1	$C_{42}H_{66}O_{14}$	22.03	$[M-H]^-$ 793.4395
33	20(*S*)-Rg_3	$C_{42}H_{72}O_{13}$	22.54	$[M-H+HCOOH]^-$ 829.4987
34	20(*R*)-Rg_3	$C_{42}H_{72}O_{13}$	22.65	$[M-H+HCOOH]^-$ 829.4972
35	Rk_1	$C_{42}H_{70}O_{12}$	23.90	$[M-H+HCOOH]^-$ 811.4854
36	Rg_5	$C_{42}H_{70}O_{12}$	24.07	$[M-H+HCOOH]^-$ 811.4894

The numbers were in accordance with Figure S6.

TABLE S2 Sequences of primers used for qPCR.

Gene	Primer sequence (5’-3’)	
	Forward	Reverse
HAVCR1	ACATATCGTGGAATCACAACGAC	ACTGCTCTTCTGATAGGTGACA
LCN2	GCAGGTGGTACGTTGTGGG	CTCTTGTAGCTCATAGATGGTGC
TNF-α	GCATGATCCGCGACGTGGAA	AGATCCATGCCGTTGGCCAG
IL-6	GAGGATACCACTCCCAACAGACC	AAGTGCATCATCGTTGTTCATACA
HDAC1	TGCTCGCTGCTGGACTTAC	GTAGGGCAGCTCATTAGGGATCT
HDAC2	GTTTTGTCAGCTCTCCACGGGT	CTTGGCATGATGTAGTCCTCCAG
HDAC5	TGAGAGGCAGGCCCTTCAGT	CCTCCAGTGCCACTCCCAAC
HDAC6	ACCGGTATGACCGTGGCACT	TCCAGGGCACATTGACAGTGA
HDAC9	TGGAGCAGCAGAGGCAAGAA	TTGCCACTGCCCTTTCTCGT
GAPDH	TTCACCACCATGGAGAAGGC	GGCATGGACTGTGGTCATGA
